# The regulation of simulated artificial oro-gastrointestinal transit stress on the adhesion of *Lactobacillus plantarum* S7

**DOI:** 10.1186/s12934-023-02174-3

**Published:** 2023-09-02

**Authors:** Dawei Chen, Chunmeng Chen, Congcong Guo, Hui Zhang, Yating Liang, Yue Cheng, Hengxian Qu, Yunchao Wa, Chenchen Zhang, Chengran Guan, Jianya Qian, Ruixia Gu

**Affiliations:** 1 College of Food Science and Engineering, Yangzhou University, Yangzhou, 225127 Jiangsu China; 2 Jiangsu Key Laboratory of Dairy Biotechnology and Safety Control, Yangzhou, 225127 Jiangsu China; 3 Jiangsu Yuhang Food Technology Co., Ltd, Yancheng, 224000 Jiangsu China; 4 Yangzhou Hospital of Traditional Chinese Medicine, Yangzhou, 225127 Jiangsu China

**Keywords:** Simulated oro-gastrointestinal transit stress, Regulation, Adhesion ability, *Lactobacillus plantarum* S7

## Abstract

**Background:**

Oro-gastrointestinal stress in the digestive tract is the main stress to which orally administered probiotics are exposed. The regulation of oro-gastrointestinal transit (OGT) stress on the adhesion and survival of probiotics under continuous exposure to simulated salivary-gastric juice-intestinal juice was researched in this study.

**Results:**

*Lactobacillus plantarum* S7 had a higher survival rate after exposure to simulated OGT1 (containing 0.15% bile salt) stress and OGT2 (containing 0.30% bile salt) stress. The adhesion ability of *L*. *plantarum* S7 was significantly increased by OGT1 stress (*P* < 0.05) but was not changed significantly by OGT2 stress (*P* > 0.05), and this trend was also observed in terms of the thickness of the surface material of *L*. *plantarum* S7 cells. The expression of surface proteins of *L*. *plantarum* S7, such as the 30 S ribosomal proteins, mucus-binding protein and S-layer protein, was significantly downregulated by OGT stress (*P* < 0.05); meanwhile, the expression of moonlight proteins, such as glyceraldehyde-3-phosphate dehydrogenase (GAPDH), phosphoglycorate kinase (PGK), beta-phosphoglucomutase (PGM1), GroEL and glucose-6-phosphate isomerase (PGI), was significantly upregulated (*P* < 0.05). However, the upregulation of GAPDH, PGK, PGM1 and PGI mediated by OGT1 stress was greater than those mediated by OGT2 stress. The quorum sensing pathway of *L*. *plantarum* S7 was changed significantly by OGT stress compared with no OGT stress cells (*P* < 0.05), and the expression of *Luxs* in the pathway was significantly upregulated by OGT1 stress (*P* < 0.05). The ABC transportation pathway was significantly altered by OGT1 stress (*P* < 0.05), of which the expression of the peptide ABC transporter substrate-binding protein and energy-coupling factor transporter ATP-binding protein EcfA was significantly upregulated by OGT stress (*P* < 0.05). The glycolide metabolism pathway was significantly altered by OGT1 stress compared with that in response to OGT2 stress (*P* < 0.05).

**Conclusion:**

*L*. *plantarum* S7 had a strong ability to resist OGT stress, which was regulated by the proteins and pathways related to OGT stress. The adhesion ability of *L*. *plantarum* S7 was enhanced after continuous exposure to OGT1 stress, making it a potential probiotic with a promising future for application.

## Introduction

Lactic acid bacteria (LAB) are important probiotics in the human intestine that have important benefits to the host, such as maintaining and reestablishing the balance of intestinal microecology, inhibiting intestinal pathogenic bacterial growth, improving immunity, enhancing antioxidant capacity and assisting in lowering blood lipids by altering the local microbiota and their metabolites in the intestine [1]. However, prebiotic functions can only be maximized when LAB arrive alive to the intestine at certain levels and colonize the human gut [2].

LAB adhere to the intestinal tract according to the mucous protein layer and intestinal epithelial cells (IECs). LAB first contact the mucus protein layer after reaching the intestine, and adhesion is mainly driven by physical binding, such as self-agglutination, hydrophobic interactions, and surface charge [3]. Their interaction is conducive to the long-term colonization of LAB and helps maintain or even increase the thickness of the mucus layer by regulating the expression of mucus protein; this improves the intestinal mucosal barrier and protects IECs from the invasion and adhesion of pathogenic bacteria [4–6].

The adhesion of LAB to IECs is mainly achieved by binding its surface protein, extracellular polysaccharide (EPS), lipoteichoic acid and other adhesins with specific IEC receptors [7–9]. The surface adhesion proteins mainly include S-layer proteins (Slps), mannose-specific adhesin (Msa), mucus binding protein (MUB), collagen binding protein (Cbp), enolase (Eno), glyceraldehyde-3-phosphate dehydrogenase (GAPDH), mucus adhesion-promoting protein (MapA), elongation factor thermo unstable (EF-TU), SpaC and fibronectin binding protein (FbpB) [7, 10]. In addition, as moonlighting proteins, some surface adhesion proteins may perform metabolic functions in the cell and can be transported to the cell-wall surface to regulate secondary biochemical functions[11], which include the glycolytic proteins of Eno, GAPDH, glucose-6-phosphate isomerase (PGI), phosphoglucomutase (PGM) and phosphoglycerate kinase (PGK), the protein folding and stress responses proteins of GroEL and DnaK, and the transcription and translation proteins of EF-TU, elongation factor Ts (EF-Ts) [12, 13]. EPS is a carbohydrate compound secreted outside the cell wall during LAB growth and metabolism [8] that has anionic characteristics that make the surface of the cell carry many negative charges and contributes to cell adhesion to IECs [14]. The low isoelectric point of lipoteichoic acid also endows the cell wall of LAB with anionic properties, which benefit LAB adhesion to IECs [9].

However, the adhesion ability of LAB is affected by many factors associated with digestive stress. Lysozyme in saliva can decrease the adhesion capacity of LAB by hydrolyzing the hydrophobic protein layer on the surface [15]. Exposure to gastric acid (pH 2.0–5.0) in the stomach for a long time may decrease the adhesion ability of LAB due to significant differences in intracellular and extracellular pH [16], which results in a change in the conformation of surface adherin [17]. In contrast, it has been found that the signaling molecules produced by LAB after oral and gastric digestion upregulate the expression of the adhesion genes groEL, dnaK and clpP, which are conducive to LAB adhesion to the intestine [18].

The cell wall of LAB is dissolved by pancreatin after it is digested in the gastric juices, and the adhesion ability can be decreased by bile salt downregulation of the surface protein related to adhesion and even by high concentrations of bile salt [19, 20]. However, a study also found that the damage by bile salt to LAB would be alleviated by low concentrations of bile salt changing the lipid bilayer of the cell membrane or the production of stress proteins, which could improve the adhesion ability [21, 22]. Hence, a high concentration of bile salt might reduce the adhesion ability of LAB, while a low concentration benefits the adhesion ability; however, the mechanism is not clear.

The metabolic changes to LAB in the upper level of the digestive tract may affect the physiological functions in the next level [23]; therefore, LAB benefit the body and need to tolerate a series of digestive stresses in addition to the digestive tract stress in individual sections. The purpose of this study was to screen LAB that had strong tolerance after continuous exposure to simulated salivary-gastric juice-intestinal juice 1 (containing 0.15% bile salt, OGT1) stress and simulated salivary-gastric juice-intestinal juice 2 (containing 0.30% bile salt, OGT2) stress and high adhesion to intestinal mucin and Caco-2 cells. Further, the regulation of OGT1 stress and OGT2 stress on adhesion ability was studied, and the possible mechanism was explored by using tandem mass tag (TMT) proteomics analysis technology. These findings provide a theoretical basis for the screening of probiotics with high intestinal adhesion and the development of related products and improve the efficiency of functional probiotics in production and application.

## Materials and methods

### Bacterial strains and growth stress

The *Lactobacillus plantarum* strains S2, 67, S7, 69, W198; the *Lactobacillus fermentum* strains 148, W120, m18, W26, m62, 128, m14; the *Lactobacillus rhamnosus* strains 108, m28, m10, m15; the *Lactobacillus paracasei* strains m85, m82, W12, 92; and the *Sterptococcus thermophilus* strains W191, W129, W131, W173 and W172 used in this work were provided by the Jiangsu Key Laboratory of Dairy Biotechnology and Safety Control of China and were isolated from traditional fermented foods and feces of Fengshan and Bama longevity, Guangxi Province, China. They were cultivated in de Man Rogosa and Sharpe (MRS) broth at 37 °C until cultures reached stationary phase.

### Tolerance to simulated artificial orogastrointestinal transit (OGT) stress

The simulated artificial OGT tolerance assay was performed according to Chen et al. [24] with some modifications. Briefly, LAB were centrifuged (3 000 ×g for 10 min at 4 °C) after growing in MRS until stationary phase, washed with sterile buffered saline (PBS) (pH 7.2; Sangon Biotech Co., Ltd., Shanghai, China), and resuspended (1 × 10^9^ CFU mL^− 1^) in simulated oral fluid (pH 7.0) with buffer solution containing 100 mg/L lysozyme (Sigma‒Aldrich, Munich, Germany) for 5 min at 37 °C. The mixture was centrifuged (3000 ×g for 10 min at 4 °C) and then resuspended in simulated gastric fluid (pH 3.0) with buffer solution containing 3.0 g/L pepsin (Sangon Biotech Co., Ltd., Shanghai, China). After 3 h of incubation at 37 °C, the LAB cells were centrifuged and incubated in simulated intestinal fluid (pH 8.0) with buffer solution containing 0.1% (w/v) pancreatin from porcine pancreas (Sangon Biotech Co., Ltd., Shanghai, China) and 0.15% (w/v, OGT1) or 0.3% (w/v, OGT2) oxgall bile salt (Solabo Technology Co., Ltd., Beijing, China) at 37 °C for 2 h. The viable count of LAB was assessed by the plate counting method with MRS agar in triplicate after incubation in simulated oral fluid, gastric fluid and intestinal fluid.

### Adhesion to intestinal mucin in vitro

The mucin-adhesion ability of LAB was evaluated in vitro as previously reported by Chen et al. [24] with minor modifications. Porcine gastric mucin (Type II; Macklin Biochemical Technology Co., Ltd, Shanghai, China) solution was prepared at 1 mg/mL in sterile PBS (pH 7.4), and 500 µL was bound to 24-well microtiter plates (Corning Inc., New York, USA) for 1 h at 37 °C; then, the samples were incubated overnight at 4 °C. A second incubation for 2 h at 37 °C was performed with the same volume. After washing twice with PBS, 500 µL of LAB suspension (500 µL; 10^8^ CFU/mL) was added to the wells and plates were incubated for 2 h at 37 °C. After non-adhered cells were removed by washing with PBS 3 times, well-adhered cells were treated with 500 µL of a 5 mL/L Triton X-100 (Sigma‒Aldrich, Munich, Germany) solution for 30 min at 37 °C, and fluid was used to assess the viable count of LAB by the plate counting method with MRS agar in triplicate. The adhesion rate was estimated using the following formula:

$$\text{Adhesion rate }\left(\%\right)=\frac{\text{CFU/mL after adhesion}}{\text{CFU/mL before adhesion}}\times 100\%$$

### Adhesion to Caco-2 cells in vitro

Caco-2 cells (Procell CL-0050, Procell Life Science & Technology Co., Ltd., China) were grown in Modified Eagle’s Medium (MEM, Life Technologies, Inc., Maryland, USA) supplemented with 20% fetal bovine serum (FBS, Clark bioscience, Virginia, USA), 1% nonessential amino acids (NEAA, Life Technologies, Inc., Maryland, USA), 1% pyruvate (Life Technologies, Inc., Maryland, USA), and 1% GlutaMAX (Life Technologies, Inc., Maryland, USA) and incubated at 37 °C with 5% CO_2_. Caco-2 cells (2 × 10^5^ cells/mL) were seeded in 24-well microtiter plates and incubated until they formed a steady monolayer. The adhesion assay of LAB was performed as previously described by Chen et al. [24]. The method used to calculate the adhesion rate was the same as that of intestinal mucin.

### Key adhesins of the LAB cell surface

LAB before and after OGT stress were washed twice with PBS; centrifuged at 3000 ×g for 10 min at 4 °C; and then resuspended in 5 mol/L LiCl (Solarbio Science & Technology Co., Ltd., Beijing, China) solution, 50 mmol/L NaIO_4_ (Sinopharm Chemical Reagent Co., Ltd) solution, and 2% (w/w) bovine serum albumin (Sangon Biotech Co., Ltd., Shanghai, China) solution at 37 °C and 200 rpm/min for 30 min to remove the surface proteins, EPS, and lipoteichoic acid [25]. LAB suspensions were washed and resuspended in PBS (1 × 10^8^ CFU/mL) after key surface adhesins were removed and added to the Caco-2 cell monolayer to test the adhesion rate [24].

### Transmission electron microscopy (TEM) observation

LAB before and after OGT stress were observed by TEM (FEI Tecnai G2 spirit, Thermo Fisher Scientific Inc., MA, USA) according to Zhu et al. [26]. After LAB pellets were postfixed in 5% glutaraldehyde at 4 °C for 24 h, samples were washed with PBS and then postfixed in 1% osmic acid in a dark environment for 2 h. The samples were dehydrated in a graded series of ethanol solutions (10, 30, 70, 80, 90, 95, 100%) after washing with PBS. The dehydrated samples were embedded in spurr low-viscosity embedding resin for 48 h. Ultrathin sections were prepared on copper grids and poststained with 2% uranyl acetate and lead citrate. Sections were then examined by TEM, and the surface thickness of the cells was analyzed by TEM particle size statistical software.

### Protein isolation and digestion

LAB before and after OGT stress were centrifuged at 5000 × g for 5 min (4 °C) and then washed 3 times with PBS (pH 7.2) for protein extraction as described by Zhu et al. [27]. SDT (4% SDS, 100 mM Tris-HCl, 1 mM DTT, pH 7.6) buffer was used for protein extraction, and the amount of protein was quantified with the BCA Protein Assay Kit (Bio-Rad, USA). Protein digestion was performed according to filter-aided sample preparation as described by Wisniewski [28]. The digested peptides of each sample were desalted on a C18 column (Empore™ SPE Cartridges C18 (standard density), Sigma) and reconstituted in 40 µL of 0.1% (v/v) formic acid after concentration by vacuum centrifugation.

### Tandem mass tag (TMT) labeling and LC–MS/MS analysis

A 100 µg peptide mixture of each sample was labeled using iTRAQ reagent according to the manufacturer’s instructions (Thermo Scientific, MA, USA) as described by Tian [29]. LC‒MS/MS analysis was carried out by coupling an Easy nLC system 1200 (Thermo Scientific, Bremen, Germany) and a Q Exactive plus (Thermo Scientific, Bremen, Germany) as previously reported by Bo [30]. Briefly, peptide samples were transported to a trap column (Thermo Scientific Acclaim PepMap100, 100 μm × 2 cm, nanoViper C18, Thermo Scientific, MA, USA) and separated on an analytical column (Thermo Scientific EASY column, 75 μm × 10 cm, 3 μm, C18: Thermo Scientific, MA, USA), which was then eluted using a gradient of 0.1% formic acid (A) (Thermo Scientific, MA, USA) and 80% acetonitrile (B) (Thermo Scientific, MA, USA) with a flow rate of 300 nL/min. Data collection was performed by Q Exactive plus. The full mass spectrometer (MS) operated in the positive ion mode, with a scan range of 300–1800 m/z with a mass resolution of 70,000, the automatic gain control (AGC) target value was set at 1e6, and the maximum ion injection time was 50 ms. The ten most intense peaks in the MS were fragmented with higher-energy collisional dissociation with a normalized collision energy of 30 eV and an underfill ratio of 1%; the resolution was set to 17,500.

### Protein identification and quantification and bioinformatics

The raw data were searched, identified and quantified using Mascot 2.2 (Matrix Science, London, UK) and Proteome Discoverer 1.4 software (Thermo Scientific, MA, USA) according to Lin et al. [31], and the database used in this study was UniProt_Lactobacillaceae_1259138_20210216.fasta. All data were reported based on 95% confidence for protein identification, as determined by a false discovery rate (FDR) ≤ 1%. Subsequently, the differentially expressed proteins (DEPs) with a fold change (FC) > 1.2 or < 0.83 and *P* < 0.05 were identified as significantly regulated proteins. Each protein function was identified by the gene ontology (GO) terms and classified by the GO enrichment analysis approach (http://beta.geneontology.org/). The online reference Kyoto Encyclopedia of Genes and Genomes (KEGG) was used for the systematic interpretation of DEPs (http://www.kegg.jp/kegg/pathway) [32].

### Statistical analysis

Data were analyzed by Tukey’s multiple comparison test using SPSS software version 20.0 (IBM Corp, NY, USA). Values are expressed as the mean ± standard deviation. When *P* < 0.05, the differences were considered significant.

## Results

### Survival of LAB after simulated OGT stress

The survival rate of 25 LAB was investigated after exposure to simulated oral, gastric, and intestinal stress. As shown in Table 1, the survival rates of *L. plantarum* S7, 69, 67, and W198; *L. fermentum* W120 and W26; and *L. paracasei* m82 remained above 10.02% and were significantly higher than those of the other strains after exposure to simulated OGT1 stress (*P* < 0.05). The survival rates of *L. plantarum* S7, 69, 67, W198 and *L. fermentum* m18 remained above 10.26%, which were significantly higher than those of the other strains after exposure to simulated OGT2 stress (*P* < 0.05). We also found that the survival rates of *L. rhamnous spp*. and *S. thermophilus spp*. were significantly lower than those of other strains after exposure to simulated OGT1 stress and OGT2 stress, respectively (*P* < 0.05).

**Table 1:** The survival rate of LAB after exposure to simulated OGT stress

Species	Strain	Initial viable counts (log10 CFU/mL)	Viable counts after exposuring in simulated saliva (log10 FU/mL)	Viable counts after exposuring in simulated saliva and gastric juice (log10 CFU/mL)	Survival rate after exposuring in simulated OGT1 stress/ (%)	Survival rate after exposuring in simulated OGT2stress/ (%)
*L. plantarum*	S2	9.59 ± 0.02^a^	9.56 ± 0.00^b^	9.38 ± 0.02^c^	3.45 ± 0.37^aJ^	0.25 ± 0.00^bI^
	S7	9.41 ± 0.02^a^	9.33 ± 0.08^ab^	9.23 ± 0.04^b^	32.69 ± 4.40^aD^	13.42 ± 2.46^bB^
	69	9.97 ± 0.01^a^	9.90 ± 0.03^b^	9.37 ± 0.00^c^	20.53 ± 1.28^aE^	12.24 ± 0.53^bB^
	67	9.06 ± 0.02^a^	9.09 ± 0.01^a^	8.94 ± 0.00^b^	172.72 ± 20.45^aA^	78.63 ± 5.61^bA^
	W198	9.46 ± 0.03^b^	9.57 ± 0.03^a^	9.60 ± 0.01^a^	68.80 ± 5.26^aB^	78.89 ± 7.01^aA^
*L. fermentum*	148	9.10 ± 0.00^a^	9.03 ± 0.01^b^	8.99 ± 0.02^b^	8.44 ± 0.06^aG^	0.02 ± 0.00^bN^
	W120	9.56 ± 0.06^a^	9.28 ± 0.00^b^	9.26 ± 0.00^b^	35.24 ± 0.58^aD^	0.45 ± 0.02^bH^
	m18	9.26 ± 0.00^a^	9.18 ± 0.01^b^	9.15 ± 0.02^b^	5.19 ± 0.30^bI^	10.26 ± 0.09^aC^
	W26	9.02 ± 0.02^a^	8.97 ± 0.02^b^	8.80 ± 0.05^b^	48.95 ± 1.90^aC^	8.65 ± 0.05^bD^
	m62	9.35 ± 0.00^a^	9.34 ± 0.02^ab^	9.30 ± 0.03^b^	6.90 ± 0.06^aH^	1.25 ± 0.12^bF^
	128	9.35 ± 0.00^a^	9.30 ± 0.00^b^	9.19 ± 0.14^b^	6.38 ± 1.45^a HI^	0.58 ± 0.06^bG^
	m14	9.37 ± 0.11^a^	9.14 ± 0.02^b^	9.13 ± 0.01^b^	8.22 ± 0.35^aG^	1.26 ± 0.02^bF^
*L. rhamnous*	108	9.72 ± 0.22^ab^	9.88 ± 0.01^a^	9.71 ± 0.02^b^	7.36 ± 0.80^aH^	0.04 ± 0.00^bM^
	m28	9.67 ± 0.03^a^	9.54 ± 0.02^b^	9.18 ± 0.20^c^	0.89 ± 0.06^aL^	0.18 ± 0.00^bJ^
	m10	9.35 ± 0.05^a^	9.34 ± 0.07^a^	9.33 ± 0.03^a^	3.05 ± 0.30^aJ^	< 0.001^b^
	m15	9.40 ± 0.03^a^	9.43 ± 0.04^a^	9.17 ± 0.07^b^	< 0.001	< 0.001
* L. paracasei*	m85	8.82 ± 0.02^ab^	8.87 ± 0.04^a^	8.76 ± 0.04^b^	0.41 ± 0.07^aM^	0.16 ± 0.01^bK^
	m82	8.97 ± 0.02^a^	8.87 ± 0.05^b^	8.79 ± 0.00^c^	10.02 ± 0.12^aF^	2.52 ± 0.24^bE^
	W12	9.14 ± 0.01a	9.18 ± 0.06^a^	9.08 ± 0.06^a^	2.67 ± 0.20^aK^	2.11 ± 0.30^bE^
	92	9.29 ± 0.01^a^	9.28 ± 0.02^a^	9.14 ± 0.02^b^	0.19 ± 0.01^aN^	0.08 ± 0.00^bL^
*S. thermophilus*	W191	8.30 ± 0.01^b^	8.38 ± 0.00^a^	8.36 ± 0.03^a^	< 0.001	< 0.001
	W129	8.28 ± 0.00^b^	8.34 ± 0.00^a^	8.28 ± 0.07^ab^	< 0.001	< 0.001
	W131	8.11 ± 0.02^b^	8.26 ± 0.00^a^	8.08 ± 0.03^b^	< 0.001	< 0.001
	W173	8.48 ± 0.03^a^	8.50 ± 0.00^a^	8.33 ± 0.02^b^	< 0.001	< 0.001
	W172	8.65 ± 0.02^a^	8.36 ± 0.00^b^	8.41 ± 0.03^b^	< 0.001	< 0.001

OGT1, simulated oral fluid, gastric fluid and intestinal fluid (w/v, 0.15% oxgall bile salt); OGT2, simulated oral fluid, gastric fluid and intestinal fluid (w/v, 0.30% oxgall bile salt). Different lowercase letters in the same row denote significant differences in the viable counts or the survival rate of the same strain (*P* < 0.05). Different capital letters in the same column denote significant differences in the survival rate among strains (*P* < 0.05). The results are expressed as the mean ± SD (*n* = 3)

### Adhesion rate of LAB to mucins and Caco-2 cells

**Fig. 1:**
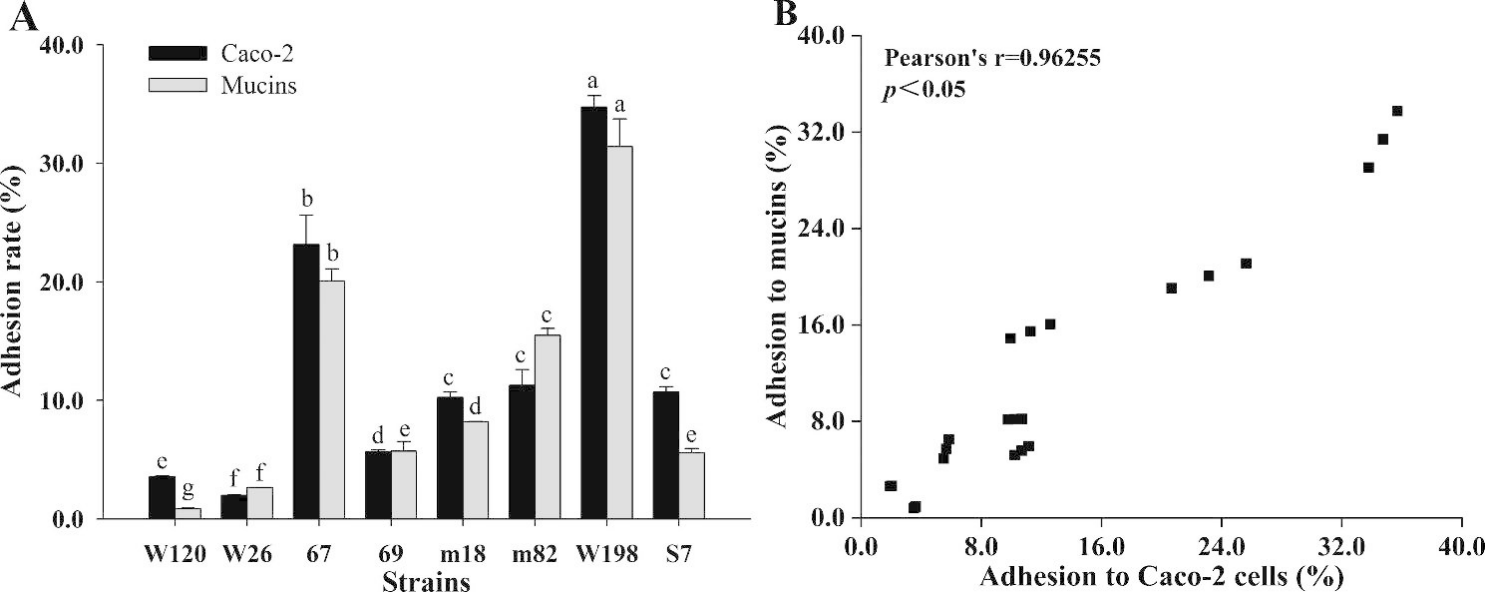
Adhesion ability of LAB. **A** is the adhesion rate of LAB to mucins and Caco-2 cells. **B** is the correlation between adhesion to Caco-2 cells and mucins. Different letters indicate significant differences in adhesion rates to Caco-2 cells and mucins (*P* < 0.05)

The adhesive ability of LAB with strong resistance to OGT stress was tested by mucins and Caco-2 cells. The adhesion rates of *L. plantarum* 67, W198, S7; *L. fermentum* m18; and *L. paracasei* m82 to Caco-2 cells were all greater than 10% and significantly higher than those of the other 4 strains (*P* < 0.05; Fig. 1A). The adhesion rates of *L. plantarum* 67, W198 and *L. paracasei* m82 to mucins were all greater than 15.47% and significantly higher than those of the other 5 strains (*P* < 0.05; Fig. 1A). At the same time, we found an interesting phenomenon in that strains with a high adhesion rate to Caco-2 cells also had a high adhesion rate to mucins; thus, there was a positive correlation (*P* < 0.05; Fig. 1B). Therefore, the adhesion rate to Caco-2 cells was used to measure the adhesive ability of LAB in a later study.

### Adhesion rate of LAB after exposure to simulated OGT stress

**Fig. 2:**
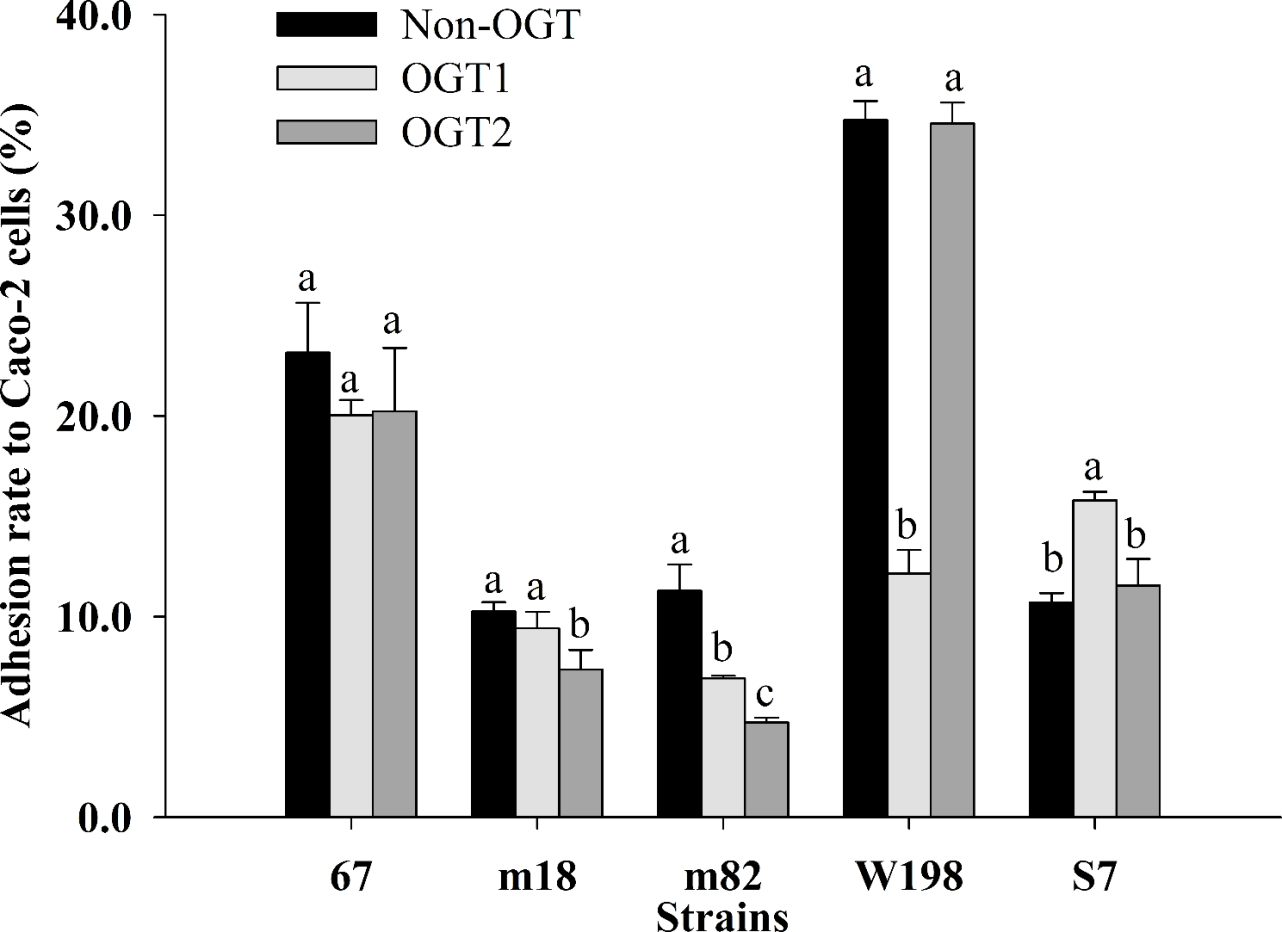
The effect of OGT stress on the adhesion rate of LAB. Different letters indicate significant differences in the adhesion rates of the strains (*P* < 0.05)

LAB with high adhesion rates were tested after exposure to simulated OGT1 and OGT2 stress. As shown in Fig. 2, OGT1 and OGT2 stress did not significantly influence the adhesion rate of *L. plantarum* 67 (*P >* 0.05), while that of *L. paracasei* m82 was significantly decreased (*P* < 0.05). The adhesion rate of *L. plantarum* W198 was significantly decreased after exposure to simulated OGT1 stress (*P* < 0.05), and that of *L. fermentum* m18 was significantly decreased by OGT2 stress (*P* < 0.05). Notably, the adhesion rate of *L. plantarum* S7 was significantly increased by OGT1 stress (*P* < 0.05) and was also increased by OGT2 stress, although not significantly (*P >* 0.05).

### Key adhesins of *L. plantarum* S7 after exposure to simulated OGT stress

**Fig. 3:**
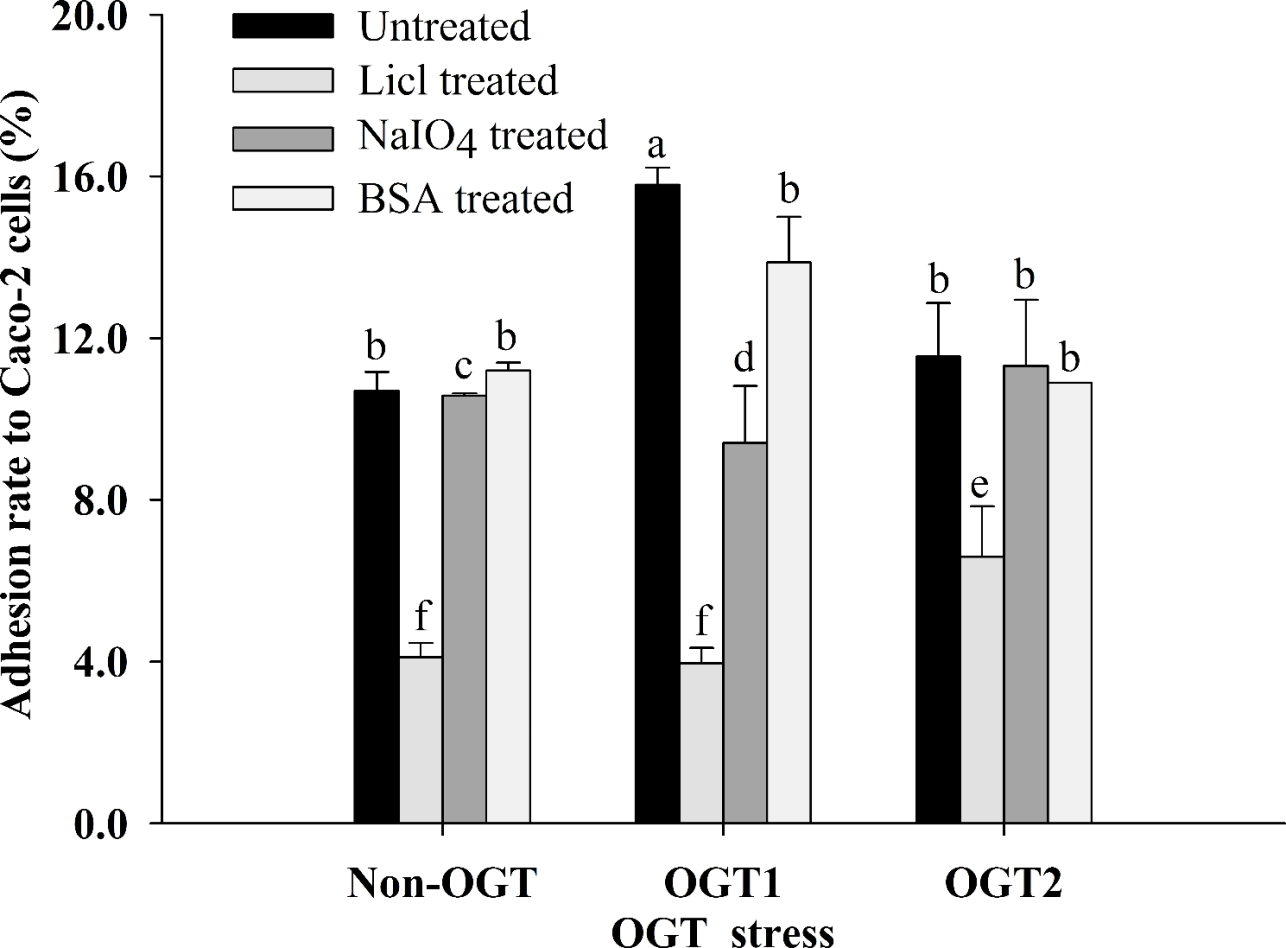
The effect of OGT stress on the key adhesins of *L. plantarum* S7. Different letters indicate significant differences in the adhesion rates of *L. plantarum* S7 after exposure to simulated OGT stress (*P* < 0.05)

The adhesion rate of *L. plantarum* S7 was significantly decreased after LiCl and NaIO_4_ treatments compared with other treatments before exposure to simulated OGT stress (*P* < 0.05; Fig. 3). The adhesion rate of *L. plantarum* S7 was significantly decreased after LiCl and NaIO_4_ treatments compared with other treatments after exposure to simulated OGT1 stress (*P* < 0.05; Fig. 3); meanwhile, the adhesion rate was significantly decreased after LiCl treatment under OGT2 stress (*P* < 0.05; Fig. 3). Therefore, surface protein and EPS may be the key adhesins of *L. plantarum* S7 under OGT1 stress, and the surface protein may be the key adhesin under OGT2 stress.

### Thickness of the cell surface of *L. plantarum* S7 after exposure to simulated OGT stress

**Fig. 4:**
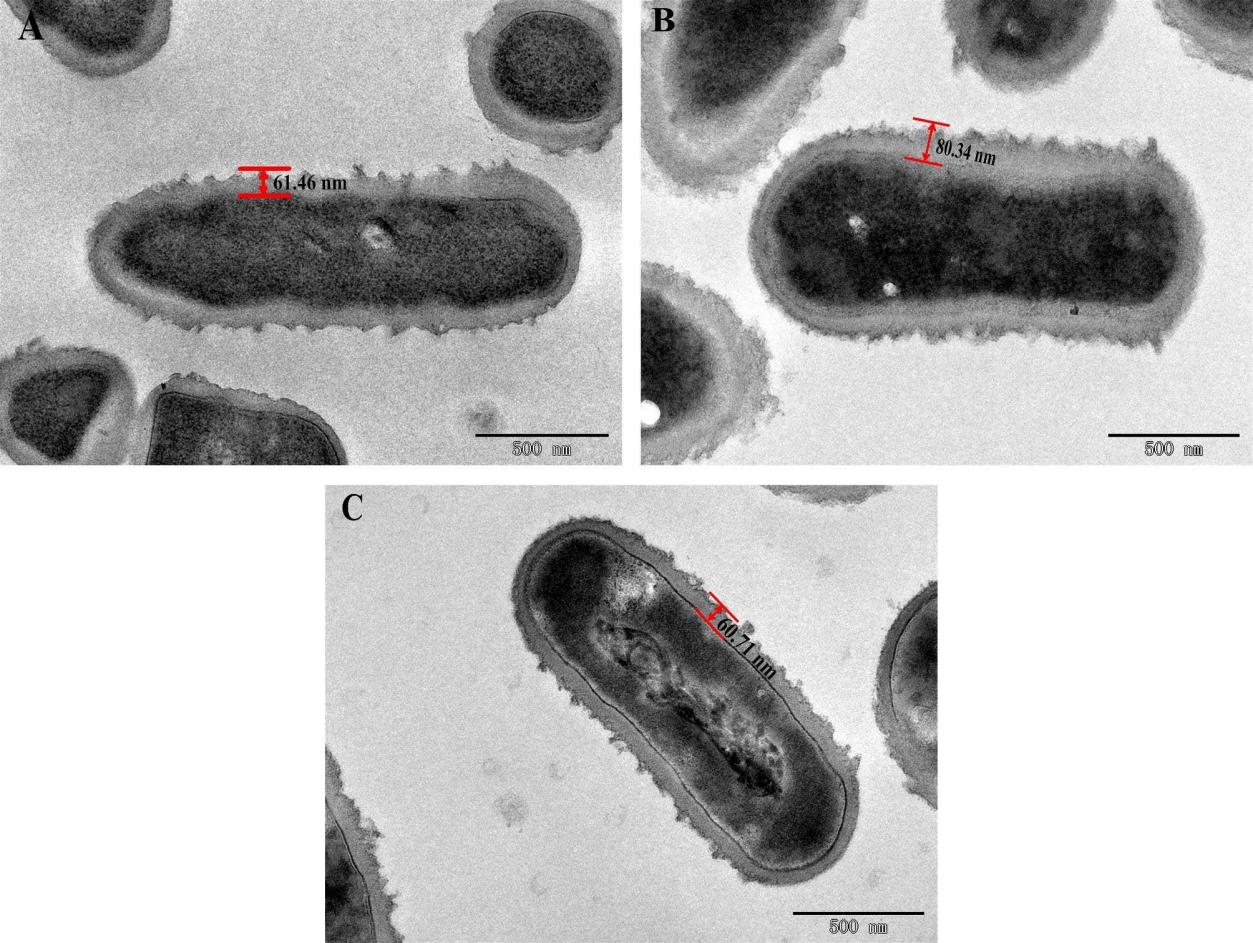
The effect of OGT stress on the cell surface thickness of *L. plantarum* S7. **A**, With non-OGT stress; **B**, With OGT1 stress; **C**, With OGT2 stress

As shown in Fig. 4, the effect of OGT stress on the shape of *L. plantarum* S7 cells was small. The thickness of the cell surface of *L. plantarum* S7 was 61.46 nm before OGT stress, and it increased to 80.34 nm after exposure to simulated OGT1 stress. OGT2 stress had little effect on the thickness of the cell surface, which was 60.71 nm. It was suggested that *L. plantarum* S7 was stimulated to secrete more substances to protect itself from the harmful OGT1 stress conditions.

### Primary data analysis of TMT results

#### DEPs in *L. plantarum* S7 after exposure to simulated OGT stress

**Fig. 5:**
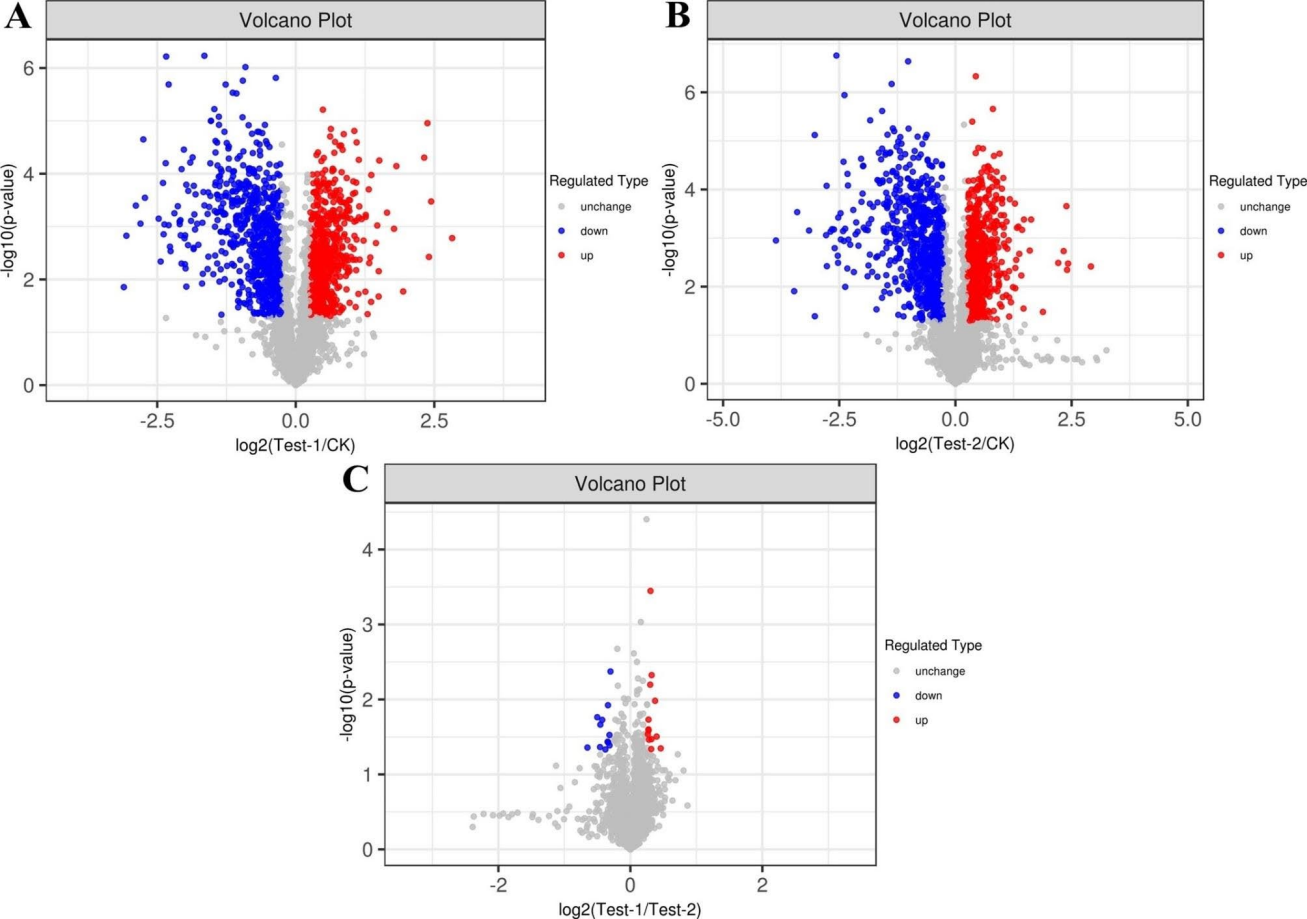
Volcano plot of *L. plantarum* S7 DEPs in response to simulated OGT stress. **A**, T/C group; **B**, S/C group; **C**, T/S group

In total, 1372 proteins of *L. plantarum* S7 were significantly differentially expressed after exposure to simulated OGT1 stress (T group) compared with non-OGT stress cells (C group; *P* < 0.05; Fig. 5A); of these DEPs, 674 proteins were upregulated and 698 proteins were downregulated (*P* < 0.05; Fig. 5A). In total, 1319 proteins were significantly differentially expressed after exposure to simulated OGT2 stress (S group) compared with the C group (*P* < 0.05; Fig. 5B); of these DEPs, 601 proteins were upregulated and 718 proteins were downregulated. Meanwhile, 13 proteins were upregulated and downregulated in the T group compared with the S group (*P* < 0.05; Fig. 5C).

The DEPs were analyzed and shown in Table 2; this group mainly included chaperone proteins, ABC transporter proteins, moonlighting proteins, ribosomal proteins, and surface.

adhesion proteins.

**Table 2:** Some DEPs of *L. plantarum* S7 in response to simulated OGT stress

Accession	Protein Name	Gene name	FC (P < 0.05)		
			T/C	S/C	T/S
A0A6G9Q5C5	EF-Ts	*tsf*	1.36	1.27	
A0A0R2CFF9	Eno	*rplL*	2.57	3.69	
A0A0R1FLN4	Eno	*eno*	0.75	0.70	
A0A1 × 1FCR4	Eno	*eno*		1.32	
A0A556UBI6	GAPDH	*gap*	2.12	1.99	
A0A2J6NNH9	GAPDH	*gap*	2.07		
A0A0R2BG78	GAPDH	*FC84_GL001279*	0.46	0.46	
A0A0L0RJR9	GAPDH	*LDI10_02755*	0.32	0.27	
A0A2S9VN17	MUB	*C6Y09_10355*	0.28	0.23	
A0A7H4UK75	MUB	*SN13T_2864*	0.70	0.68	
A0A7H4UIK4	MUB	*SN13T_2293*	0.24	0.20	
A0A2S9VJ12	MUB	*C6Y10_16405*		0.81	
A0A1W7QJ71	PGK	*pgk*	1.33		
A0A0R2FIL6	PGK	*pgk*	1.37		
G0M2V0	PGM	*LPENT_01109*	1.20		
A0A0R2L5R9	PGI	*pgi*	0.23	0.22	
A0A0R1ME61	PGI	*pgi*	1.21		1.21
A0A1Z5IF51	PGI	*pgi*	1.24		
A0A0R1YRA3	PGI	*pgi*	1.25		
A0A0R2B1P9	PGI	*pgi*	1.27		
A0A5P0ZG74	PGI	*pgi*	1.30		
A0A2R3JUY8	PGI	*pgi*	1.35	1.32	
A0A241RSC7	PGI	*pgi*	1.39		
A0A494S936	PGI	*pgi*	1.43		
A0A656YBF7	PGI	*FC93_GL001516*	1.56	1.62	
A0A2A7QFI3	S-layer protein	*CP368_08905*	0.65	0.59	
A0A5D0JPP9	S-layer protein	*FXE12_11685*	0.33	0.36	
A0A0R2MK70	Cell surface protein	*hsp2*	1.74	1.92	
A0A2K9HYX9	Cell surface protein	*SN13T_2231*		0.81	
G0M5Z3	Cell surface protein	*SN13T_0949*	0.74	0.73	
A0A2S9VZQ3	Cell surface protein	*BB562_03225*	0.75	0.71	
A0A2S9VVI8	Cell surface protein	*C6Y09_00950*	0.71	0.70	
A0A2K9HYY5	Cell surface protein	*BB562_02955*	0.73	0.65	
A0A2S9W5I2	Cell surface protein	*C6Y08_11780*	0.67	0.60	
A0A7H4UIN0	Cell surface protein	*SN13T_2292*	0.15	0.18	
A0A2K9I7E0	Cell surface protein	*SN13T_0861*	0.51	0.52	
T5JI40	Cell surface protein	*BB562_02535*	1.48		
A0A7H4UJ42	Cell surface protein, membrane-anchored	*hsp1*	1.81	1.55	
A0A7H4UES7	Cell surface protein, YbbR-like family	*IV64_GL001758*		1.22	
A0A7H4UEJ8	Cell surface protein, LPXTG-motif cell wall anchor	*SN13T_0861*	0.34	0.28	
A0A7H4UE12	Cell surface hydrolase, LPXTG-motif cell wall anchor	*SN13T_0671*	0.19	0.15	
A0A494S2N7	Protein GrpE	*grpE*		1.28	
A0A7H4UF27	Co-chaperonin GroES	*groES*	1.89	1.85	
A0A7H4UF22	Chaperonin GroEL	*groEL*	1.53	1.51	
A0A076L0K5	GroEL (Fragment)	*groEL*	1.25	1.29	
F6IUH0	Lipoprotein, peptide binding protein OppA homolog	*LPE_01240*	1.46	1.48	
A0A7H4UHQ4	Putative oligopeptide ABC transporter, oligopeptide-binding protein OppA	*oppA2*	1.95	1.77	
A0A0R1TCJ9	Lipoprotein, peptide binding protein OppA-like protein	*FC17_GL000707*	1.70	1.64	
A0A7H4UEY5	Lipoprotein, peptide binding protein OppA-like protein	*SN13T_0987*	1.47	1.50	
A0A0R2MV82	ABC transporter, substrate-binding protein, family 5	*IV56_GL002274*	2.54	2.21	
G0M1I4	Oligopeptide ABC transporter, substrate binding protein	*LPENT_00701*	1.88	1.70	
W6T9Z9	Peptide ABC transporter substrate-binding protein	*LFAB_05760*	2.28	1.97	
A0A3M6LPK6	Peptide ABC transporter substrate-binding protein	*D6U19_00650*	1.25	1.37	
A0A151G3Q3	Peptide ABC transporter substrate-binding protein	*AVR82_13925*	1.87	1.50	1.25
A0A4Z0JAZ0	ABC transporter substrate-binding protein	*EGT51_04530*	2.84	2.67	
A0A494S5A6	S-ribosylhomocysteine lyase	*LuxS*	1.21		
F9URP8	PTS system, cellobiose-specific EIIA component	*pts20A*	1.22		
A0A7H4UFB0	PTS system, mannose-specific EIIAcomponent	*pts10A*	1.40	1.49	
A0A7H4UJS7	PTS system, N-acetylglucosamine /galactosamine-specific EIIA component	*pts19A*	0.61	0.56	
A0A7H4UH03	Beta-phosphoglucomutase (PGM1)	*pgmB1*	1.96	1.90	
A0A0R1U020	Chaperone protein DnaK	*dnaK*	3.52	3.09	
A0A0R1WED9	Chaperone protein DnaK	*dnaK*	2.82	2.14	
A0A0R2L0N0	Chaperone protein DnaK	*dnaK*	1.41	1.41	
W6T7C4	Chaperone protein DnaK	*dnaK*	1.30		
A0A5P0ZLK4	Chaperone protein DnaK	*dnaK*		1.29	
A0A0C1PPD7	Chaperone protein DnaK	*dnaK*		1.25	
A0A0R1N5Z2	Chaperone protein DnaK	*dnaK*		1.30	
A0A5Q2NX50	Chaperone protein DnaK	*dnaK*	1.31		
A0A0R2L1T9	30 S ribosomal protein S1	*rpsA*	1.39	1.32	
A0A0R2H741	30 S ribosomal protein S2	*rpsB*	0.70		
A0A2I9CK53	30 S ribosomal protein S2	*rpsB*	0.72	0.67	
A0A0R1M822	30 S ribosomal protein S12	*rpsL*	0.71	0.60	
A0A426DAP1	30 S ribosomal protein S12	*rpsL*	0.69	0.66	
A0A5Q2NZ70	30 S ribosomal protein S21	*rpsU*	0.48	0.41	
A0A2R3JSP0	30 S ribosomal protein S3	*rpsC*	0.75		
A0A512PKJ4	30 S ribosomal protein S13	*rpsM*	0.25	0.19	
A0A0R2DEV0	50 S ribosomal protein L1	*rplA*	0.77	0.75	
A0A0R2MIH7	50 S ribosomal protein L6	*rplF*	0.64	0.64	
A0A0R1HZ67	50 S ribosomal protein L14	*rplN*	2.51	2.76	
A0A660DXH6	50 S ribosomal protein L10 [Lactobacillus sp.]	*MUDAN_MDHGFNIF_00600*	4.99	5.24	
Q88Z52	50 S ribosomal protein L31 type B	*rpmE2*	1.32	1.31	
A0A0R2DQ84	50 S ribosomal protein L7/L12	*rpmF*	0.71	0.74	
A0A6N9I1Z5	50 S ribosomal protein L32	*rpsL*	0.52	0.50	
Q88WS9	50 S ribosomal protein L32	*rpmF*	0.45	0.41	
A0A7H4UE85	50 S ribosomal protein L30	*rpmD*	0.68	0.58	
A0A0R2AFB3	50 S ribosomal protein L30	*rpmD*	0.67	0.59	
A0A2S9W7C9	3-oxoacyl-[acyl-carrier-protein] synthase 3	*fabH*	0.78	0.72	
A0A7H4UJ90	D-lactate dehydrogenase	*SN13T_2512*	1.92	2.00	
A0A0R2F637	Triosephosphate isomerase (TIM)	*tpiA*	1.32		
A0A199QFI9	Triosephosphate isomerase	*tpiA*	1.27	1.27	
A0A2S9W6H0	Dihydrolipoyl dehydrogenase (DLD)	*lpdA*	1.34	1.33	
A0A2K9I2Y5	Energy-coupling factor transporter ATP-binding protein EcfA	*ecfA*		1.20	
G0M278	Energy-coupling factor transporter ATP-binding protein EcfA	*ecfA*	1.59	1.52	
F6IUS8	Alkaline phosphatase superfamily protein	*LPE_01361*	1.26	1.23	
A0A0F3RP94	Clp protease ClpX	*VC81_13110*	1.20		
A0A1L6H9E0	ATP-dependent Clp protease ATP-binding subunit	*BTW26_02810*	1.45		
A0A4Q0VIJ3	ATP-dependent Clp protease ATP-binding subunit	*DXH47_09740*	1.42		
A0A3R8J6Z0	ATP-dependent Clp protease ATP-binding subunit	*D1831_09805*	1.35		
W6T8J2	ATP-dependent Clp protease ATP-binding protein	*LFAB_05835*	1.30		
W6T906	ATP-dependent Clp protease ATP-binding protein	*LFAB_04560*	0.71		
A0A7H4UMJ7	ATP-dependent Clp protease ATP-binding subunit ClpX	*clpX*	0.63		
A0A7H4UJU1	Small heat shock protein	*hsp2*	0.73	0.75	
A0A2Z6DRC0	60 kDa heat shock protein (Fragment)	*hsp60*	1.28	1.28	
Q70BV3	60 kDa chaperonin (Fragment)	*hsp60*	1.44	1.39	
Q6TCD3	GroEL (Fragment)	*hsp60*	1.76		

#### GO enrichment analysis

**Fig. 6:**
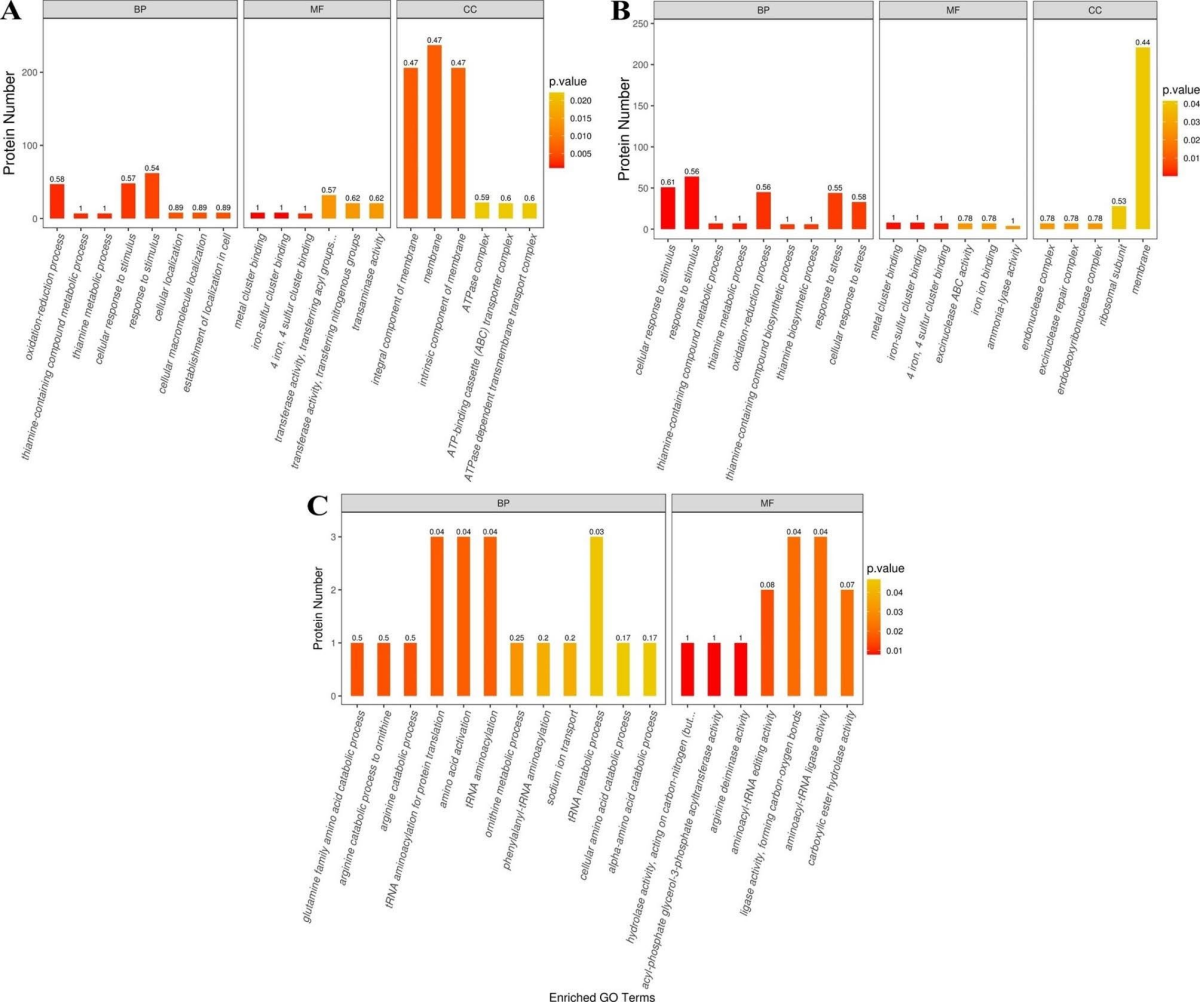
GO enrichment analysis of *L. plantarum* S7 DEPs in response to simulated OGT stress (top 20). **A**, T/C group; **B**, S/C group; **C**, T/S group

After GO function analysis, we found that the response to stimulus (GO: 0050896), the cellular response to stimulus (GO: 0051716), and the oxidation‒reduction process (GO: 0055114) in the biological process (BP) category were extremely significantly enriched after *L. plantarum* S7 was exposed to simulated OGT1 and OGT2 stress (*P* < 0.01; Fig. 6A and B); these categories contained a large number of DEPs. In addition, the response to stress (GO: 0006950) and the cellular response to stress (GO: 0033554) in the BP category were also extremely significantly affected by OGT2 stress and contained a large number of DEPs (*P* < 0.01; Fig. 6B).

The DEPs of metal cluster binding (GO: 0051540), iron-sulfur cluster binding (GO: 0051536), and 4 iron, 4 sulfur cluster binding (GO: 0051539) in the Molecular Function (MF) category were extremely significantly changed after *L. plantarum* S7 was exposed to simulated OGT1 and OGT2 stress (*P* < 0.01; Fig. 6A and B). The integral component of membrane (GO: 0016021), the membrane (GO: 0016020), and the intrinsic component of membrane (GO: 0031224) in the cellular component (CC) were extremely significantly enriched after *L. plantarum* S7 was exposed to simulated OGT1 stress (*P* < 0.01; Fig. 6A); this categories contained a large number of DEPs.

In addition, hydrolase activity, acting on carbon-nitrogen (but not peptide) bonds in linear amidines (GO: 0016813), acyl-phosphate glycerol-3-phosphate acyltransferase activity (GO: 0043772) and arginine deiminase activity (GO: 0016990) in the MF category were extremely significantly changed after *L. plantarum* S7 was exposed to simulated OGT1 stress when compared to that following OGT2 stress (*P* < 0.01; Fig. 6C).

#### KEGG enrichment analysis

**Fig. 7:**
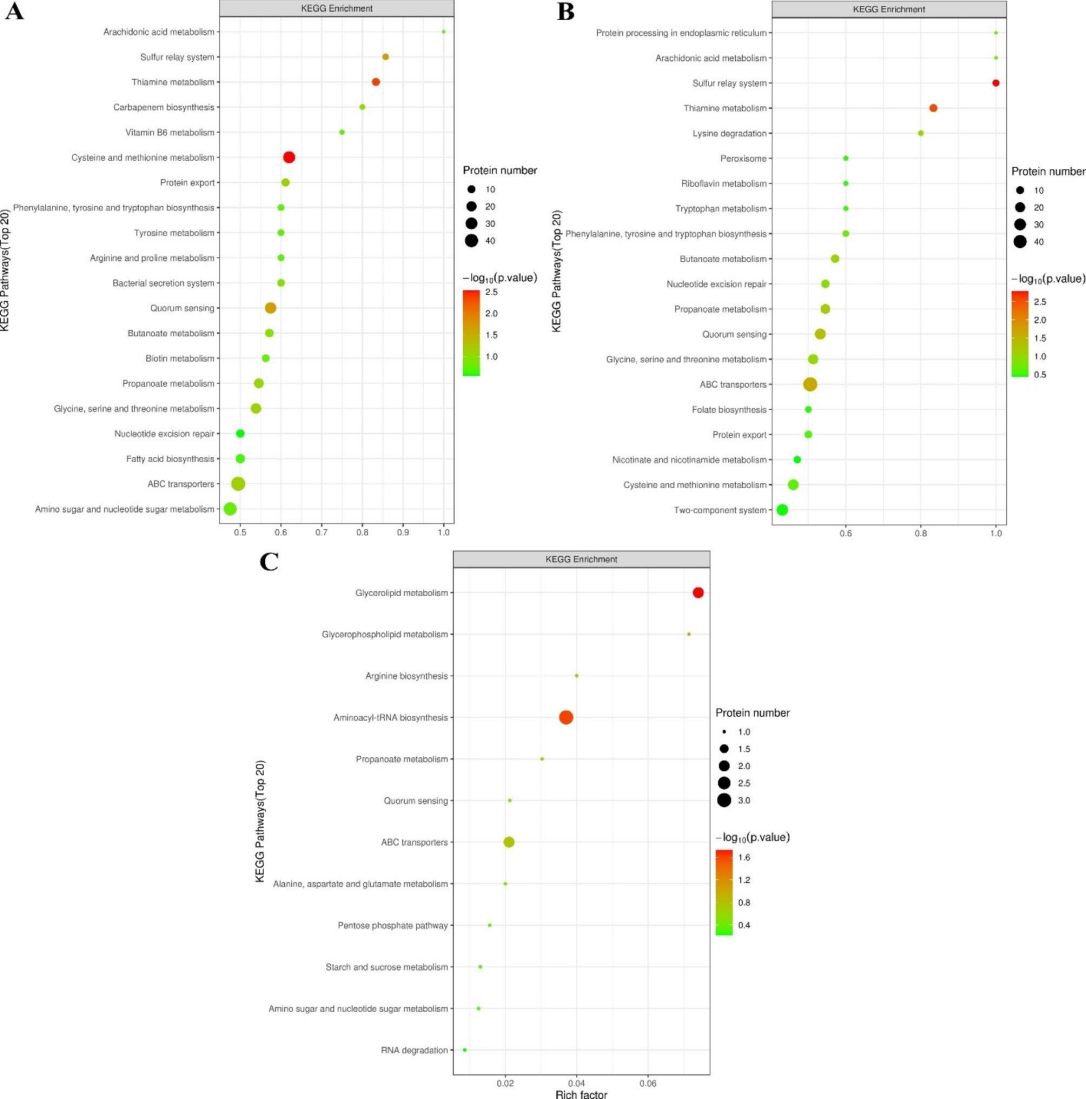
KEGG pathway enrichment scatter plot of DEPs of *L*. *plantarum* S7 in response to simulated OGT (top 20). **A**, T/C group; **B**, S/C group; **C**, T/S group

The DEPs were further subjected to KEGG pathway analysis. As shown in Figs. 7A and 31 DEPs were associated with cysteine and methionine metabolism (ko00270; P = 0.0029), 10 DEPs were associated with thiamine metabolism (ko00730; P = 0.0041), 27 DEPs were associated with quorum sensing (ko02024; P = 0.021), and 6 DEPs were associated with the sulfur relay system (ko04122; P = 0.024) after *L. plantarum* S7 was exposed to simulated OGT1 stress. After exposure to simulated OGT2 stress, 7 DEPs were associated with the sulfur relay system (P = 0.0053), 10 DEPs were associated with thiamine metabolism (P = 0.0076), 25 DEPs were associated with quorum sensing (p = 0.019), and 48 DEPs were associated with ABC transporters (ko02010; P = 0.036; Fig. 7B). When compared with OGT2 stress, 2 DEPs were associated with glycerolipid metabolism (ko00561; P = 0.018), and 3 DEPs were associated with aminoacyl-tRNA biosynthesis after *L. plantarum* S7 was exposed to simulated OGT1 stress (ko00970; P = 0.025; Fig. 7C).

## Discussion

LAB may stimulate various protective mechanisms to resist adverse stress, such as the gastric acid environment and intestinal bile, when passing through the digestive tract. LAB maintained high activity when exposed to simulated saliva in our study (Table 1), which may be attributed to the upregulation of *hsp* gene expression by oral stress [33] and the high survival rate following simulated stomach digestion (Table 1); this may indicate that the pH balance inside and outside the cell was improved by regulating the proton transfer of membrane ATPase, glutamic acid decarboxylase, arginine deaminase and urease after being stressed by the stomach environment [34]. However, the survival rate of the strains decreased significantly after continuous exposure to OGT stress (*P* < 0.05; Table 1), which suggested that the continuous stresses of OGT, such as lysozyme, pepsin, pH, trypsin and bile salt, had a greater impact on the vitality of strains during the digestion process.

It was found that the metabolic pathways of LAB, such as glycolysis/gluconeogenesis, population sensing, ABC transport, molecular chaperone, nucleotide transport and metabolism, and amino acid biosynthesis, participate in adaptation to acid, bile salt and other harmful environments during OGT [35].

### The effect of OGT stress on the glycolysis/gluconeogenesis pathway of *L. plantarum* S7

A proteomic study found that 71 and 57 DEPs were enriched in the glycolysis/.

gluconeogenesis pathway (ko00010) of *L. plantarum* S7 after exposure to OGT1 and OGT2 stress; although this pathway was not significantly changed, it did have the most enriched DEPs, indicating that this pathway plays an important role in the process of *L. plantarum* S7 resistance to OGT stress. The expression of GAPDH, PGK, triosephosphate isomerase (TIM), PGM, PGI, Eno and D-lactate dehydrogenase in the pathway was significantly upregulated after exposure to OGT1 and OGT2 stress (*P* < 0.05; Table 2); these proteins contribute to binding host epithelial components, such as mucin and extracellular matrix components, and directly bind IECs [13, 36, 37]. At the same time, the upregulation of PGK and TIM expression can help maintain the balance of carbohydrate metabolism, and the upregulation of ENO, GAPDH and TIM may increase energy and help sustain ATP-dependent processes in the *L. plantarum* S7 response to OGT stress [38–40]. These results showed that the upregulation of related DEPs in the Glycolysis/Gluconeogenesis pathway was stimulated by OGT stress to protect the carbohydrate metabolism and adhesion ability of *L. plantarum* S7; the upregulation multiples of moonlight proteins of GAPDH, PGK, PGM and PGI by OGT1 was more than that in response to OGT2 (Table 2). This may be an important reason why the adhesion of *L. plantarum* S7 after exposure to OGT1 stress was significantly higher than that of OGT2 (*P* < 0.05; Fig. 2).

### The effect of OGT stress on the quorum sensing of *L. plantarum* S7

Quorum sensing (QS) is a bacterial communication signal system that automatically secretes and releases signal molecules to achieve physiological regulation by sensing changes in the concentration of bacteria in the surrounding environment in a cell density-dependent manner [41]. The QS pathway (ko02024) of *L. plantarum* S7 changed significantly after exposure to OGT1 and OGT2 stress (*P* < 0.05; Fig. 7B), and the putative oligopeptide ABC transporter, oligopeptide-binding protein OppA, lipoprotein, peptide binding protein OppA-like protein, oligopeptide ABC transporter, substrate binding protein and ABC transporter, substrate-binding protein, family 5 in the pathway were also significantly upregulated; these are the main proteins involved in biofilm formation [42], indicating that OGT stress can promote the biofilm formation of *L. plantarum* S7 and is conducive to survival.

The Luxs/AI-2 QS system helps bacteria adapt and survive in the unfavorable environment of OGT and promotes adhesion to IECs [43, 44]. The expression of the biosynthetic protein *LuxS* in the QS pathway was also significantly upregulated after exposure to OGT1 stress but not after exposure to OGT2 stress (*P* < 0.05; Table 2); this protein is the necessary catalyst for the synthesis of the signal molecule AI-2 in the pathway. Furthermore, the loss of AI-2 reduced the adhesion ability of Lactobacillus to Caco-2 cells, while the adhesion ability was restored after adding AI-2 [45, 46]; thus, AI-2 may be an important signal molecule in the process of *L. plantarum* S7 adhesion to IECs. In addition, AI-2 can also increase the production of EPS [47], and EPS was the main adhesin of *L. plantarum* S7 after exposure to OGT1 stress but not OGT2 stress in our study (Fig. 3). This suggested that OGT1 stress could increase AI-2 synthesis and EPS production in the QS pathway more than OGT2 stress by significantly upregulating the expression of *Luxs*, which significantly increased the adhesion ability of *L. plantarum* S7 after exposure to OGT1 when compared to that after exposure to OGT2 (*P* < 0.05; Fig. 2).

### The effect of OGT stress on the nucleotide transport and metabolism of *L. plantarum* S7

Monosaccharides are transported into the cytoplasm, and the synthesis of glucose-1-phosphate, the activation and linkage polymerization of sugars, and the output of EPS are four important pathways for the synthesis of EPS [48]. The PTS system is responsible for the transport of monosaccharides. PGM1 is involved in the synthesis of glucose-1-phosphate from PGI and plays an important role in the formation of sugar nucleotides, such as UDP-glucose [49–51]. Furthermore, UDP-glucose is closely related to the synthesis of nucleoside sugar and EPS in the processes of the activation and linkage polymerization of sugars [52]. In our study, the PTS system proteins PGI and PGM1 were significantly upregulated after *L. plantarum* S7 exposure in OGT1 and OGT2 (*P* < 0.05; Table 2), indicating that OGT stress promotes EPS biosynthesis. The extent of upregulation after exposure to OGT1 conditions was greater than those after exposure to OGT2 conditions; this may have resulted in EPS being one of the main adhesions of *L. plantarum* S7 after exposure to OGT1 but not to OGT2.

The glycolide metabolism pathway (ko00561) of *L. plantarum* S7 was significantly changed after exposure to OGT1 stress compared with that after exposure to OGT2 stress. The alkaline phosphatase superfamily protein in the pathway is the main protein responsible for the synthesis of lipoteichoic acid [48] and was significantly upregulated (*P* < 0.05; Table 2), which may have enhanced the adhesion of *L. plantarum* S7 by stimulating the production of lipoteichoic acid after exposure to OGT1 stress.

### The effect of OGT stress on the ABC transporter pathway of *L. plantarum* S7

The ABC transportation system is an essential pathway for adhesion-related surface proteins through the plasma membrane and is also critical to the metabolism of nutrients and toxic molecules [49, 53], which contribute to the bacterial adaptation to changing environments and survival under OGT stress. The ABC transporter pathway (ko02010) of *L. plantarum* S7 was significantly changed after exposure to OGT2 stress, and the expression of the peptide ABC transporter substrate-binding protein involved in adhesion to gastrointestinal mucus in the pathway was also significantly upregulated (*P* < 0.05; Table 2), which may have improved the adhesion ability of *L. plantarum* S7 after exposure to OGT1 and OGT2 stress [54]. However, the upregulation OGT1 stress was 1.25 times that after OGT2 stress, which may have resulted in the adhesion ability of *L. plantarum* S7 being higher than that of OGT2 after exposure to OGT1 stress. The energy-coupling factor transporter ATP-binding protein EcfA in the pathway is responsible for the transmembrane transport of nutrients in cells [55], and the extent of upregulation of OGT2 was greater than that of OGT1 (Table 2), indicating that *L. plantarum* S7 needs more EcfA to minimize environmental damage by absorbing nutrients and increasing hazardous substance export after exposure to OGT2 stress when compared with exposure to OGT1 stress. These findings indirectly showed that OGT2 stress is more harmful to *L. plantarum* S7 than OGT1 stress.

### The effect of OGT stress on the chaperone proteins of *L. plantarum* S7

Chaperones can alleviate molecular aggregation and the misfolding of bacterial proteins under stress; for example, the chaperones DnaK, GroEL and GroES could provide a favorable environment for protein folding, contribute to the maturation of synthesized proteins and protein repair and improve adhesion [56, 57]. The expression of the chaperone proteins DnaK, chaperonin GroEL and cochaperonin GroES of *L. plantarum* S7 was significantly upregulated after exposure to OGT1 and OGT2 stress (*P* < 0.05; Table 2), indicating that the activity of *L. plantarum* S7 might be maintained by significantly upregulating the proteins involved in repairing acid damage or assisting in the folding of newly synthesized proteins [35]. The expression of the Clp protein ClpX was significantly upregulated after exposure to OGT1 but not after exposure to OGT2 (*P* < 0.05; Table 2), and the Clp protein can both help maintain the quality of cell proteins and help chambers counter the harsh effects of bills [58, 59]; this may be an important factor contributing to the observation that *L. plantarum* S7 survival was significantly higher after exposure to OGT1 stress than after exposure to OGT2 stress (*P* < 0.05; Table 1).

DLD was enriched in the pyruvate metabolism (ko00620); propanoate metabolism (ko00640); glycolysis/gluconeogenesis (ko00010); tryptophan metabolism (ko00380); biosynthesis of cofactors (ko01240); glycine, serine and threonine metabolism (ko00260); glyoxylate and dicarboxylate metabolism (ko00630); valine, leucine and isoleucine degradation (ko00280); and lysine degradation and citrate cycle (TCA cycle; ko00020) pathways and was significantly upregulated (*P* < 0.05; Table 2) after *L. plantarum* S7 exposure to OGT1 and OGT2 stress. DLD might play a role in the transport of solutions into and out of the cell, which may contribute to the cytoplasmic transport of chaperonin GroEL, chaperone protein DnaK, EF-Ts, ENO and PGI to the cell-wall surface and be conducive to amino acid synthesis which is required for cell growth [60]; thus, DLD might support the adhesion and survival of *L. plantarum* S7.

### The effect of OGT stress on the surface proteins of *L. plantarum* S7

The ribosome pathway (ko03010) was not significantly changed after *L. plantarum* S7 exposure to OGT stress, but 64 and 67 DEPs were enriched in the pathway. The 30 S ribosomal proteins (S2, S12, S13, S3, S21) and 50 S ribosomal proteins (L1, L6, L7/L12, L30, L32) in the pathway were significantly downregulated (*P* < 0.05; Table 2); these changes may have contributed to favoring the maintenance and regeneration of proteins in *L. plantarum* S7 over the biosynthesis of new proteins to reduce the amount of energy used and utilize energy effectively, thereby aiding in survive under OGT stress [56]. As surface proteins, 30 S ribosomal proteins also regulate adhesion to the intestine [61]. Therefore, the survival of *L. plantarum* S7 was maintained by significantly downregulating the expression of surface proteins in the ribosome pathway (*P* < 0.05; Table 2); meanwhile, the adhesion ability of cells was decreased after exposure to OGT1 and OGT2 stress.

MUB, sortase A, S-layer protein, and cell surface protein are also important adhesion proteins on the surface of LAB [62]; however, they may be hydrolyzed by trypsin and acid in the simulated OGT condition [63], resulting in their expression being significantly downregulated (*P* < 0.05; Table 2). This indicated that OGT stress has a great impact on the expression of surface proteins and reduces the adhesion ability of *L. plantarum* S7.

## Conclusion

The survival and adhesion of LAB were greatly impacted and regulated by continuous exposure to OGT1 and OGT2 stress. However, *L. plantarum* S7 had strong resistance to OGT stress, and survival and adhesion could be regulated by increasing the expression of proteins related to protein synthesis, carbohydrate metabolism, biofilm formation, harmful substance export and their KEGG pathways. In addition, the expression of moonlight proteins, proteins related to the synthesis of lipoteichoic acid and EPS, was significantly upregulated after *L. plantarum* S7 was exposed to OGT1 stress (*P* < 0.05), which increased the thickness of the surface material of the cell and increased the survival rate and adhesion ability of *L. plantarum* S7 after exposure to OGT1 stress (containing 0.15% bile salt) compared with that after OGT2 stress (containing 0.30% bile salt).

## Data Availability

All materials described within this manuscript, and strains are available on request.
